# Cracking the Crack Dance: A Case Report on Cocaine-induced Choreoathetosis

**DOI:** 10.7759/cureus.1981

**Published:** 2017-12-22

**Authors:** Naureen Narula, Faraz Siddiqui, Nakul Katyal, Nithya Krishnan, Michel Chalhoub

**Affiliations:** 1 Internal Medicine, Staten Island University Hospital; 2 Pulmonary and Critical Care, Staten Island University Hospital; 3 Neurology, University of Missouri Columbia

**Keywords:** cocaine complications, involuntary movement, neuro critical care

## Abstract

Movement disorders represent one of the less common presentations of cocaine toxicity observed in clinical practice. Given the magnitude of crack cocaine use, it is vital to understand the underlying pathogenesis. We present a case of a patient who clinically exhibited cocaine-induced choreoathetosis. The diagnosis was confirmed after ruling out all other organic causes of de novo choreoathetoid movement. This case highlights the association of cocaine with choreoathetoid movements. We propose a preliminary understanding of the underlying pathogenesis, which may help intensivists better recognize this uncommon phenomenon.

## Introduction

Movement disorders represent one of the less common presentations of cocaine toxicity observed in clinical practice. Though there is little clinical or literature evidence documenting cocaine-induced hyperkinesis, the street names of “crack-dancing” suggest that it may be more common than physicians recognize [1]. Given the magnitude of crack cocaine use and availability, it is vital to understand the underlying pathogenesis. Cocaine-induced hyperkinesis, includes de novo tics, dystonia, essential-like tremor, myoclonus, and chorea [1]. Such movement disorders are known to occur in cocaine abuse, dependence, and withdrawal [1]. A rare presentation amongst this spectrum is cocaine-induced choreoathetosis. This presentation was first reported by Kamath and Bajaj [2]. They documented videographic evidence of a patient exhibiting severe choreiform movements affecting the limbs, neck, and orolingual region [2]. In this study, we present a case of a patient who clinically exhibited cocaine-induced choreoathetosis. The diagnosis was confirmed after ruling out all other organic causes of de novo choreoathetoid movement.

## Case presentation

**Figure 1:**
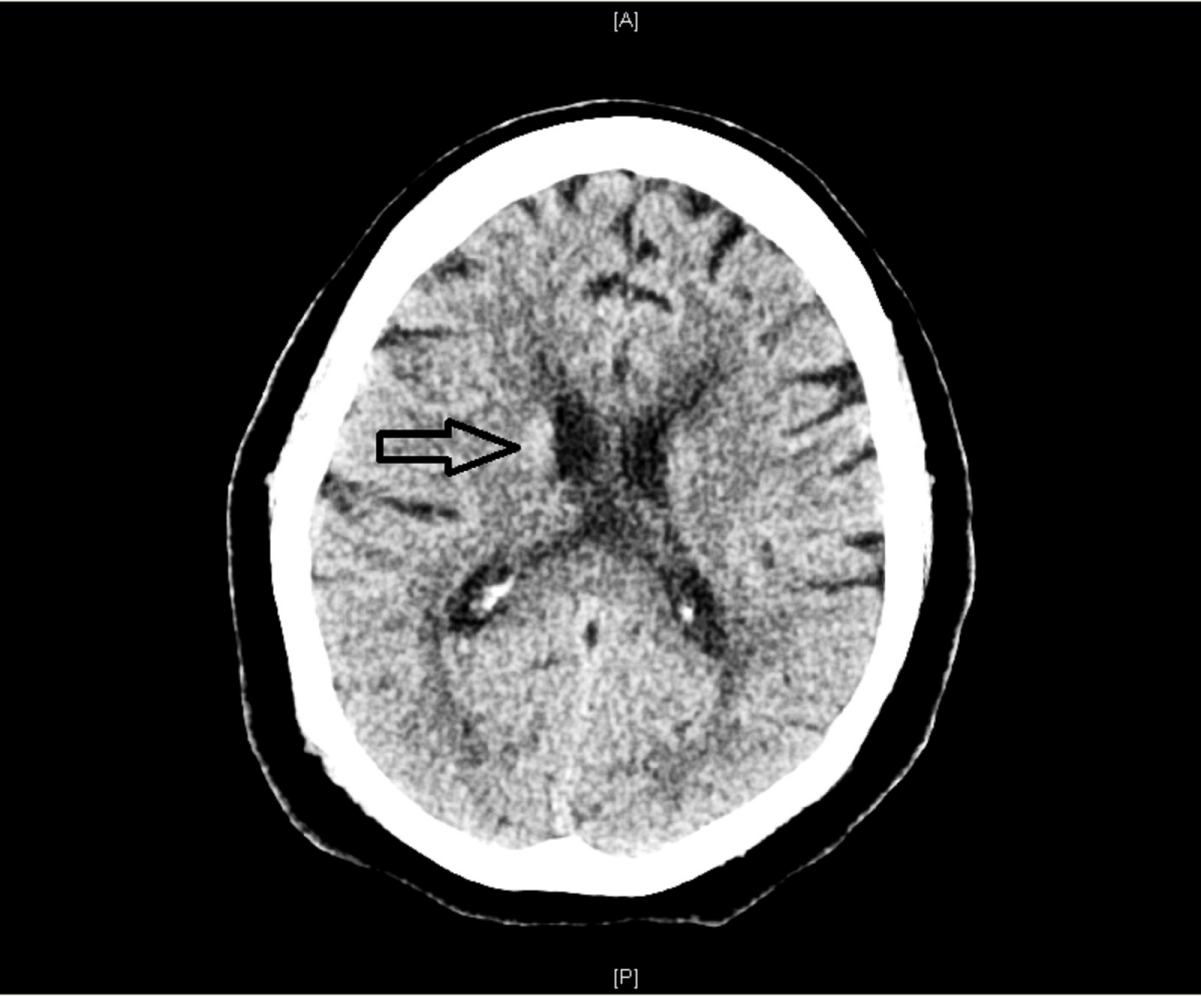
Computed tomography of the head

The patient's history was provided by a reliable historian. A 69-year-old female presented to the emergency department (ED) with chief complaints of experiencing difficulty maintaining balance, along with dry mouth and decreased appetite of one day duration. She recalled having increased urinary frequency from the past one month. The patient reported having a history of type II diabetes mellitus and being noncompliant with medication use. Her vital signs in the ED revealed a blood pressure of 113/76 mmHg, a respiratory rate of 14 per min, a heart rate of 86 beats per min and a temperature of 36.2°C. On physical examination, she exhibited severe choreiform movements affecting the extremities, more pronounced in the upper extremities and in the orolingual region. She denied using any antipsychotic medications. However, she admitted to using crack cocaine two days prior to presentation in light of some life stressors. Laboratory investigation showed a blood glucose level of 660 mg/dl and an anion gap of 17. A working diagnosis of nonketotic hyperosmolar hyperglycemia was established, and she was admitted to the intensive care unit (ICU). A computed tomography (CT) head scan showed an area of hyperlucency around the putamen (Figure 1).

The patient received 1500 ml of normal saline, 10 units of regular insulin followed by insulin drip, 1 gm of intravenous (IV) Keppra and 5 mg of IV Diazepam. Subsequently, her metabolic condition improved and she was downgraded to the neurology floor. However, she continued having involuntary choreiform movements involving bilateral extremities affecting the left more than the right. On day two of the admission, a 12-items anchored Abnormal Involuntary Movement Scale (AIMS) [3] was performed to assess the severity of the patient’s movement disorder which pointed towards moderate to severe involuntary movement disorder. Complete blood count (CBC), basic metabolic panel (BMP), salicylate levels, acetaminophen levels, serum copper, and ceruloplasmin levels came back as negative. The urine toxicology screen was positive for cocaine. After ruling out all possible neurological diagnoses, the only pertinent information remaining was the patient’s recent history of crack cocaine use. Given the similar instances reported in literature, a diagnosis of cocaine-induced choreoathetosis was established. The involuntary movements gradually improved over the next few days without any medical intervention, with the lower extremities improving first, followed by the upper extremities. The patient was discharged after 12 days of hospital stay.

## Discussion

In this report, we highlighted a rare phenomenon of cocaine-induced choreoathetosis or crack dancing. Choreoathetoid movements are self-limiting and characteristically involve orofacial and limb musculature and can last for up to several days [4, 5]. Preexisting organic brain disease has been reported as a potential risk factor for the development of this disorder [2, 4]. Because the movements are self-limited, affected individuals are not likely to seek medical care [6]. Thus it is difficult to estimate the actual prevalence of this phenomenon [6]. The underlying pathophysiology involves the blockade of dopamine reuptake by cocaine leading to high availability of dopamine at the synaptic cleft, which can trigger choreoathetoid movements [4]. While the pathophysiology differs substantially from long-term degeneration of dopaminergic tracts in the basal ganglia as seen in Huntington’s disease, cocaine-induced choreoathetoid movements are clinically indistinguishable from those seen in Huntington’s disease [4]. This may explain the transitory nature of the choreiform movements with cocaine use. In cocaine abusers, the inability to downregulate dopamine concentration may cause recurrence of these symptoms [4]. The role of D1 dopamine receptors in cocaine-induced dopamine signaling has been demonstrated both cellularly and behaviorally [6, 7]. A number of electrophysiological studies have identified alterations in dopamine neurotransmission at the receptor level within the mesoaccumbens neurons following chronic cocaine exposure [6, 7].

The 12-item Abnormal Involuntary Movement Scale (AIMS) can be used to assess clinical presentations associated with choreiform movements [3]. It is often used to record the occurrence of dyskinesia in patients receiving neuroleptic medications [3]. AIMS is a 12-item anchored scale that is clinician administered and scored. Items 1-4 assess orofacial movements, 5-7 deal with extremity and truncal dyskinesia, and 8-10 deal with global severity as judged by the examiner and the patient’s awareness and discomfort associated with the movements [3]. Items 11-12 discuss problems with teeth or dentures because these can lead to a misdiagnosis of dyskinesia [3]. The guidelines regarding the management of hyperkinetic movements associated with cocaine toxicity are still not clear owing to the relative rarity of this condition. Supportive care is the mainstay of treatment with attention to maintenance of the airway, vital signs, and hydration status. Neuroleptics, which are central dopamine antagonists, can be used for treatment. Chlorpromazine is one such medication that can be administered parenterally. Haloperidol is a potent dopamine receptor blocker with relatively fewer side effects than chlorpromazine. Benzodiazepines can also be used to reduce the sympathetic drive.

Our case highlights the rare presentation of choreoathetosis associated with cocaine abuse and now adds to the limited number of cases in literature describing this uncommon phenomenon.

## Conclusions

Crack cocaine use has significantly increased throughout the United States. Given the magnitude of cocaine abuse, a possible association with choreoathetosis cannot be overlooked. Through this report, we have proposed a preliminary understanding of the underlying pathogenesis, which may help intensivists better recognize this uncommon phenomenon.
